# Large- and small-scale population structure of *Xanthomonas oryzae* pv. *oryzicola*, a bacterial pathogen of rice

**DOI:** 10.1128/aem.01121-25

**Published:** 2025-09-08

**Authors:** Anne Sicard, Sara C. D. Carpenter, Amadou Diallo, Shivranjani Baruah, Cheick Tekete, Lazeni Konate, Ibrahim Keita, Hinda Doucoure, Phuong Duy Nguyen, Quyen Le Cao, Soungalo Sarra, Mamadou Dembele, Charlotte Tollenaere, Lucie Poulin, Hamidou Tall, Laurence Blondin, Valérie Verdier, Ralf Koebnik, Sylvain Zougrana, Harinjaka Raveloson, Lionel Gagnevin, Geoffrey Onaga, Sebastien Cunnac, Christian Vernière, Issa Wonni, Ousmane Koita, Boris Szurek, Adam Bogdanove, Mathilde Hutin

**Affiliations:** 1 Univ Montpellier, IRD, CIRAD, INRAE, Institut Agro, Plant Health Institute of Montpellier 740764, Montpellier, France; 2 INRAE, UMR SVQV, Université de Strasbourg 27083, Colmar, France; 3 Plant Pathology and Plant-Microbe Biology Section, School of Integrative Plant Science, Cornell University 517685, Ithaca, New York, USA; 4 Laboratoire de Biologie Moléculaire Appliquée, des Techniques et des Technologies de Bamako, Faculté des Sciences et Techniques, Universite des Sciences 225803, Bamako, Mali; 5 Department of Molecular Pathology, Agricultural Genetics Institute, Hanoi, Vietnam; 6 Centre Régional de Recherche Agronomique de Niono, Institut d'Economie Rural 612900, Bamako, Mali; 7 Institut Sénégalais de Recherches Agricoles 206826, Kolda, Senegal; 8 INERA, Institut de l'Environnement et de Recherches Agricoles 317957, Bobo-Dioulasso, Burkina Faso; 9 Centre Régional de Recherche du FOFIFA/DP SPAD, Antsirabe, Madagascar; 10 Université de Vakinankaratra, Antsirabe, Madagascar; 11 National Agricultural Research Organization 128532, Kampala, Uganda; 12 Africa Rice Center 102689, Bouake, Côte d'Ivoire; The University of Tennessee Knoxville, Knoxville, Tennessee, USA

**Keywords:** rice, molecular epidemiology, *Xanthomonas oryzae*, bacterial leaf streak

## Abstract

*Xanthomonas oryzae* pv. *oryzicola* is a pathogen of rice responsible for bacterial leaf streak, a disease that can cause up to 32% yield loss. While it was first reported a century ago in Asia, its first report in Africa was in the 1980s. Since then, it has been discovered in several countries of both East and West Africa. In this study, we used a combination of genomics and genotyping tools to shed light on its global and local diversity and population structure with a focus on West Africa. Our findings group *X. oryzae* pv. *oryzicola* strains into three clades, an Asian, an East African-Asian, and a West African clade, and provide evidence of several introduction events from Asia being at the origin of outbreaks in East Africa and probably in West Africa. Our results further highlight the role of human activities in the local spread of this pathogen and the possible role of several wild rice species and weeds as reservoirs. Finally, our results globally support a clonal evolution of *X. oryzae* pv. *oryzicola*. However, coinfection of plants with distinct strains of *X. oryzae* pv. *oryzicola*, observed in Mali and Senegal, may facilitate some degree of genetic exchange.

**IMPORTANCE:**

West Africa has faced a rapid expansion of rice cultivation with importation of rice varieties mostly from Asia, and rice now constitutes 37% of the cereal consumed in the region. The bacterial pathogen *Xanthomonas oryzae* pv. *oryzicola* (*Xoc*) is causing bacterial leaf streak and threatening rice production in West Africa. Little is known about the pathogen’s reservoirs and its modes and routes of dissemination. We used genome sequencing and tandem repeat sequences to describe large- and small-scale population structure and molecular epidemiology. Our results support the role of rice seed trade in the local and global spread of *Xoc*. This study further suggests different introduction events from Asia to both East and West Africa. We describe local natural dispersal events with some clonal diversification and the possible role of wild rice and weed species as reservoirs. Overall, our results indicate that weed management and the enforcement of phytosanitary measures on rice seeds could help control the spread of bacterial leaf streak.

## INTRODUCTION

Rice is the first cultivated cereal for human consumption and one of the main crops in Africa and Asia. Its importance as a staple food has increased over the last decades, particularly in West Africa, partly driven by the 2008 food price crisis (1). Two domestication events have led to its cultivation: the domestication of *Oryza sativa* in Asia more than 10,000 years ago (2) and the domestication of the African rice species, *Oryza glaberrima,* in Northern Mali 3,500 years ago (3). Today, *O. sativa*, which provides better yields, is widely grown in Africa (3). The global expansion of *O. sativa* may have favored the propagation of pests infecting it. This crop is indeed prone to infection by a high number of pathogens such as fungi, bacteria, nematodes, and viruses, some of which are seed-transmitted (4).

In this study, we focus on *Xanthomonas oryzae* pv. *oryzicola* (*Xoc*), which causes bacterial leaf streak (BLS). BLS can reduce rice yields by up to 32% (5). Colonizing the intercellular spaces of the parenchyma, this pathogen can be spread by wind, rain, irrigation water, insects, and seeds (6). Since BLS has a lower incidence and generally lower severity on rice than bacterial leaf blight caused by *X. oryzae* pv. *oryzae* (5), *Xoc* has so far been much less studied. BLS was first reported in the Philippines in 1918 but was mistakenly referred to as bacterial blight for several decades. It was only recharacterized in 1957 in China (6). *Xoc* can be easily distinguished phenotypically and now genetically from the pathovar *oryzae* (6, 7). It is now widespread in tropical and subtropical Asia (6). Its presence on the African continent was reported in the early 1980s in West Africa and Madagascar and more recently in several countries of East Africa (8–14). Recently, sequencing of an herbarium rice leaf sampled in Madagascar in 1931 allowed us to date the first description of BLS in Africa back 50 years (15).

Only a few epidemiological studies of *Xoc* have been carried out using molecular markers. These studies were based on restriction fragment length polymorphism, multilocus sequence analysis, multilocus variable number of tandem repeats analysis (MLVA), and type 3 effector gene diversity (16–20). MLVA is a highly discriminant genotyping method used to characterize populations and study the epidemiology of organisms with low genetic diversity, such as *X. oryzae*, for which little sequence polymorphisms are observed when sequencing a few genes (17, 21). MLVA is based on the size polymorphism of short tandem repeats (TR) at different locations in the genome, which is mainly generated by DNA replication slippage (22). In terms of evolution, variable number of TR (VNTR) loci evolve faster than other portions of the genome, allowing to study populations at small geographic and temporal scales (21). In 2015, Poulin et al. (17) applied an MLVA-16 scheme on a collection of 152 *Xoc* strains from West Africa and Asia. They were able to separate *Xoc* strains into two groups, an Asian group and a West African (WA) group, with the exception of two strains from China that were genetically related to strains from Mali and Burkina Faso (17). In another important study, whole-genome sequencing of 10 *Xoc* strains, including three from West Africa, showed that the content of type three effectors allows to distinguish Asian strains from WA ones (20). However, the strict conservation of some type three effectors raises questions about the relationship between WA *Xoc* and Asian strains (20). These studies do not include the East African strains, preventing us from understanding the global distribution, diversity, and dissemination routes of the pathogen. We recently demonstrated in a study of 20 genomes of *Xoc*, including the first five East African genomes sequenced, that *Xoc* strains form three groups, the third one comprising the five East African strains and some Asian ones (15). Here, we have built a collection of 399 strains of *Xoc* from 12 countries to shed light on epidemiological characteristics of the disease from both a global and a local perspective, with a focus on West Africa. We first sequenced 27 new genomes and combined these with 21 published genomes to perform whole-genome sequence comparisons and identify phylogenetic branches. We then applied to the entire collection of 399 strains a MLVA to estimate more finely the genetic relationships. The results presented in this study provide a clear picture of the local distribution and diversity of this pathogen in West Africa and a better understanding of the global epidemiology of BLS.

## MATERIALS AND METHODS

### Bacterial strains and culture conditions

A collection of 399 *Xoc* strains coming from 12 countries was used in this study. It is the largest collection of strains genotyped for this pathogen so far. The collection includes 106 strains already genotyped by Poulin et al., but we re-genotyped these in our MLVA pipeline to avoid bias from using a different sequencing platform (13, 16, 17). The strains were collected between 1964 and 2020 from symptomatic leaves of at least eight different plant species, a few plants not having been identified. Most of the strains were collected from West Africa, namely, from Senegal (57 strains), Mali (134 strains), and Burkina Faso (106 strains) between 2003 and 2020. Strain metadata is presented in Table S1. Strains were isolated on PSA medium (10 g/L peptone, 10 g/L sucrose, 1 g/L glutamic acid, and 16 g/L bacto agar) supplemented with cycloheximide (50 mg/L), kasugamycin (20 mg/L), and cephalexin (40 mg/L). Cultures grown from single colonies were stored at −80°C in 15% glycerol until use. To ensure that a single clone was genotyped, bacteria were streaked again before use, and single colonies were resuspended in 100 µL distilled sterile water before being lysed for MLVA, or were used to inoculate liquid cultures for DNA extraction and genome sequencing.

### Genome sequencing, *de novo* assembly, and phylogenetic analysis

Using single-molecule real-time sequencing (Pacific Biosciences, HiFi sequencing), the genome sequences of 23 *Xoc* strains from five countries in East Africa and three in West Africa (Table S2) were generated at the Icahn School of Medicine Genomics Core facility at Mount Sinai (New York, New York). Raw reads were assembled using the SMRT Link Microbial Assembly pipeline (SMRT Link v8.0.0.80529) using default settings. The complete assemblies are accessible at the National Center for Biotechnology Information (NCBI) under Bioproject PRJNA731279. MD-IVORY-1-C, VXO32, and VXO39 were sequenced using the Illumina Hi-Seq2500 platform (Fasteris SA, Plan-les-Ouates, Switzerland), and draft genome sequences were assembled using the Edena algorithm v3.131028 (23) (Table S2). BAI15 was sequenced using Solexa GAllx/HiSEQ and assembled with Trimmomatic v. 0.32, SGA v. 0.10.13, iMetAMOS v. 1.5, samtools v. 1.1, FastQC v. 0.10.0, Spades v. 3.1.1, idba v. 1.1.1, Pilon v. 1.8, Quast v. 2.2, and Prokka v. 1.7. The complete assemblies are accessible at the NCBI under BioProjects PRJNA257008 (MD-IVORY-1-C), PRJNA374501 (BAI15), PRJNA270507 (VXO32), and PRJNA1254927 (VXO39). A core genome alignment including all *Xoc* sequenced strains was obtained using Roary v3.13.0 (24) and was used to build a maximum likelihood (ML) tree using RAxML v.8.2.12 (25). The best-scoring tree was generated using the GTRGAMMA substitution model and 1,000 bootstrap replicates. ClonalFrameML v1.12 (26) was employed with default parameters to detect recombination events as well as to estimate the relative role of recombination over point mutations in *Xoc* evolution.

### Geographic ancestral state reconstruction

A phylogenetic tree rooted with the reference *Xoo* strain, BAI3 (27), was used to reconstruct ancestral scenarios. West Africa, East Africa, and Asia were used as possible locations for the ancestor represented at each internal node of the phylogeny. A ML method, the marginal posterior probabilities approximation, implemented in PastML (26) was used with an F81-like model to predict the ancestral node for every node.

### PCR and sequencing for MLVA

The strain collection was genotyped using the MLVA-16 scheme developed for *X. oryzae* by Poulin et al. in 2015 (17). Briefly, four multiplex PCR reactions, each containing four fluorescently labeled primer pairs each, were run to genotype each strain using the QIAGEN Multiplex PCR Kit (Qiagen, Courtaboeuf, France). PCR amplifications were performed as follows: for PCR mixes 1 and 2, 35 cycles of denaturing at 94°C for 30 s, annealing at 60°C for 1 min 30 s, and extension at 72°C for 1 min, and for PCR mixes 3 and 4, the same conditions except annealing at 64°C and 25 cycles. PCR products were diluted before being mixed with GeneScan 500 or 600 LIZ dye Size Standard and Hi-Di formamide (Applied Biosystems, Courtaboeuf, France). Capillary electrophoresis was performed in an ABI 3500 XL Genetic Analyzer (Applied Biosystems) at the GenSeq facility (UMR ISEM, CeMEB LabEx) in Montpellier, France. Two loci (G07 and G88) were removed from the subsequent analyses because of amplification issues for most of the strains. Moreover, G58 was removed because of alternate motifs of different sizes, 4 and 7 bp, and G44 was taken out since this locus is absent in *Xoc* strains. The resulting MLVA-12 scheme consists of 10 polymorphic loci and two monomorphic loci previously described as diagnostic markers for the pathovar *oryzicola* (17).

### MLVA data scoring

Fragment sizes for each locus were estimated by using GeneMapper version 4.0 (Applied Biosystems, Life Technologies, Carlsbad, CA) and converted to repeat numbers. The sizes of the flanking regions for each locus were determined by analyzing the Sanger sequences using MAI3 and BLS256 as reference strains for African and Asian *Xoc* strains, respectively. When needed, the number of repeats was rounded up to the nearest integer as previously recommended (28).

### Genetic analyses

Indices of gene and genotypic diversities were calculated using the R package poppr v.2.8.6 (29). Allelic richness and private allelic richness were calculated using a rarefaction method implemented in HP-rare (29). Linkage disequilibrium (LD) for WA populations was tested at either the village/city scale (Mali and Burkina Faso) or at the field scale (Senegal) as well as at the country scale using the index of association *r*_*d*_ from the R package poppr v.2.9.1 (29). Genetic differentiation between populations was measured using both pairwise *F*_ST_ and *R*_ST_ statistics computed by Arlequin v.3.5.2.2 (30). Phylogenetic relationships between haplotypes (allelic profiles) were depicted by minimum spanning trees using an algorithm combining global optimal eBURST and Euclidean distances implemented in the software PHYLOViZ 2.0 (31). Groups of strains differing one from another for a single TR locus, that is, single locus variant (SLV), were grouped into clonal complexes (CCs). A dendrogram with bootstrap support (1,000 bootstrap replicates) was computed using the aboot function of the R package poppr v.2.9.1 (29). The Nei distance and the unweighted pair group method with arithmetic mean algorithm were used to build this dendrogram. A tanglegram was built using the R package dendextend (32) to visualize the congruence between the MLVA-based and the core genome-based trees. Baker’s Gamma index was computed using the same package to measure the similarity between trees.

## RESULTS

### Global population structure of *Xoc* and sources of genetic variation based on whole-genome sequences

**Fig 1:**
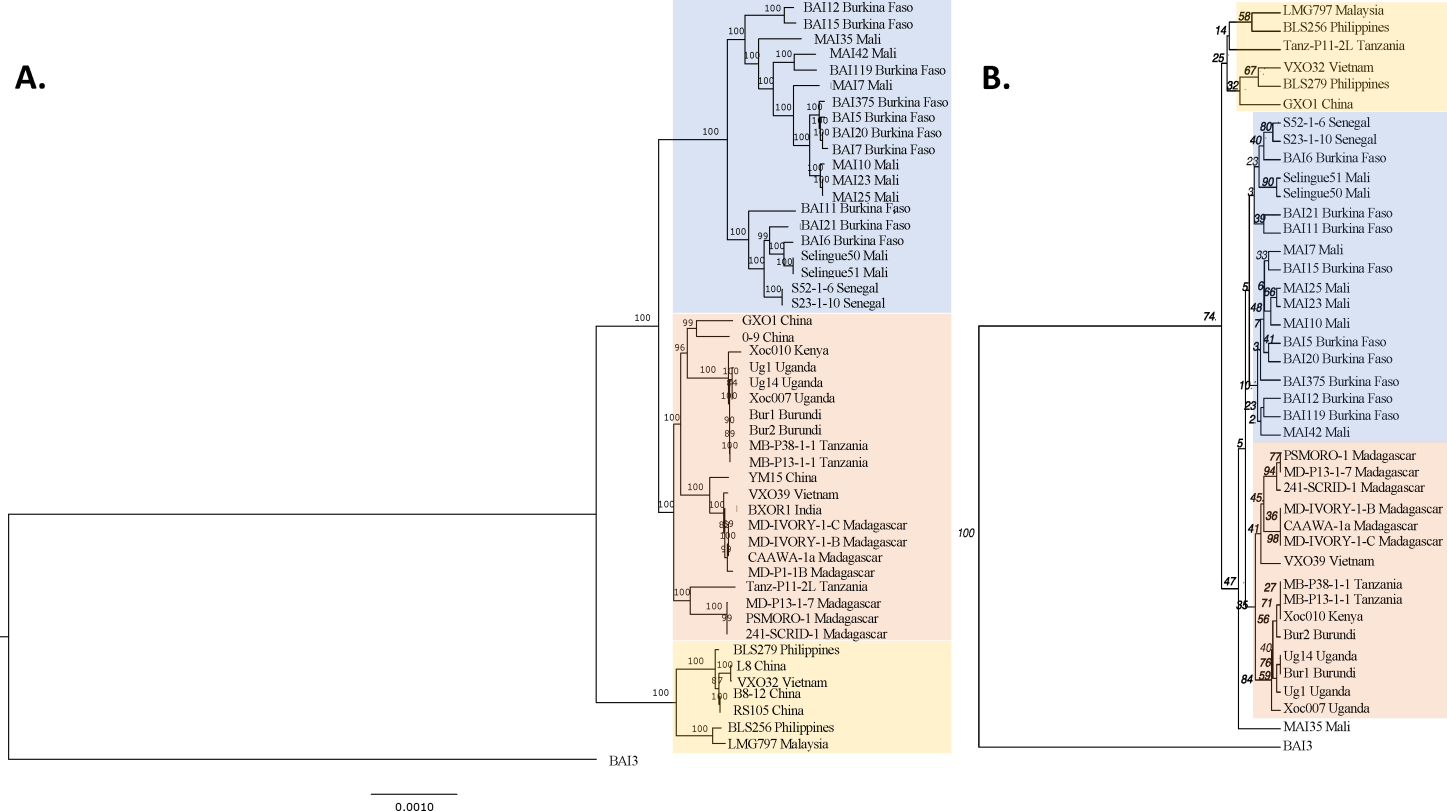
ML phylogenetic tree and dendrogram showing the relationships among a worldwide collection of sequenced *Xoc* strains. The ML phylogenetic tree (**A**) was built using the core genome alignment of 48 *Xoc* strains, with the *X. oryzae* pv. *oryzae* strain BAI3 as an outgroup, while the dendrogram (**B**) was built using the MLVA data of 41 of those *Xoc* strains. Bootstrap values show support for each tree node. The Asian clade appears in yellow, the EA-A clade in red, and the WA one in blue.

Twenty-one genomes of *Xoc* were publicly available at the time of the analyses, respectively, six and 10 genomes of WA and Asian strains, and the recently sequenced genomes of five East African strains (15). To better represent *Xoc* distribution and its worldwide genetic diversity, 27 new genomes of *Xoc* strains collected between 2004 and 2016 were sequenced. Of these, 11 originated from East Africa, 14 from West Africa, and two from Vietnam (Table S2). The average depth of coverage was 416-fold. A ML phylogenetic tree was built using this collection of 48 genomes and the African *X. oryzae* pv. *oryzae* BAI3 strain as an outgroup (Fig. 1A). Three main clades supported by high bootstrap values were observed: (i) an “Asian” clade composed of strains from the Philippines, Malaysia, China, and Vietnam; (ii) an “East African-Asian” clade composed of strains from Burundi, Uganda, Tanzania, Kenya, Madagascar, India, China, and Vietnam; and (iii) a “West African” clade comprising the strains from Mali, Burkina Faso, and Senegal (Fig. 1A).

Interestingly, three different sub-clades were found within the EA-A clade, with Asian strains in two of these sub-clades and the third clade being composed of a strain from Tanzania and strains from Madagascar. These results suggest at least two different introduction events from Asia to East Africa, one to mainland East Africa and one to Madagascar. Within the Asian clade, two sub-clades were observed, one consisting of strains from the Philippines and Malaysia and the other of strains from the Philippines, China, and Vietnam. Finally, two main sub-clades were found within the WA clade, one including strains from all three WA countries represented in the collection and the other including strains from Mali and Burkina Faso. None of the Asian or East African sequenced strains were found within the WA clade. An ancestral state reconstruction was performed to trace the ancestral history of *Xoc* and the origin of the different clades. The locations of the *Xoc* root and of the nodes of the three clades were resolved to Asia (Fig. S1) corroborating that *Xoc* originated from Asia and was then introduced to both East and West Africa.

Removing recombination did not change the tree topology. A ClonalFrameML analysis showed that the ratio of recombination over point mutation, the mean length of imports, and the average distance of the imports were respectively equal to *R*/*θ* = 0.183382, *δ* = 282.14 bp and *ν* = 0.014. Based on these results, mutations are around 5.45 times more frequent than recombination and cause overall 1.4 times more substitutions than recombination in *Xoc*.

### Genetic differentiation of populations between countries

In order to expand our analyses to a larger collection of strains, we first examined whether the different clades observed using whole-genome sequencing were also found when applying the widely used, higher-throughput typing method MLVA. The MLVA-12 scheme for *X. oryzae*, adapted from Poulin et al. (17), was used for genotyping 41 out of the 48 sequenced strains, seven sequenced strains being absent from our collection. Overall, the trees were highly congruent (Baker’s gamma coefficient 0.82), with the three clades and their relationships to each other preserved. However, differences within clades as well as two differences among clades were observed with the Tanzanian strain Tanz-P11-2L residing inside the Asian clade but with little support and the Malian strain MAI35 branching from the base of the EA-A/WA clades (Fig. 1B; Fig. S2).

Based on this overall congruence, we used MLVA to expand our genotyping study to the entire collection of 399 strains. We first checked that the number of loci used was sufficient to discriminate among the different genotypes present within the collection by computing a rarefaction curve (Fig. S3). A plateau was reached using 10 of these loci, implying that the MLVA-12 scheme is sufficient to uncover the collection’s genetic diversity.

**Fig 2:**
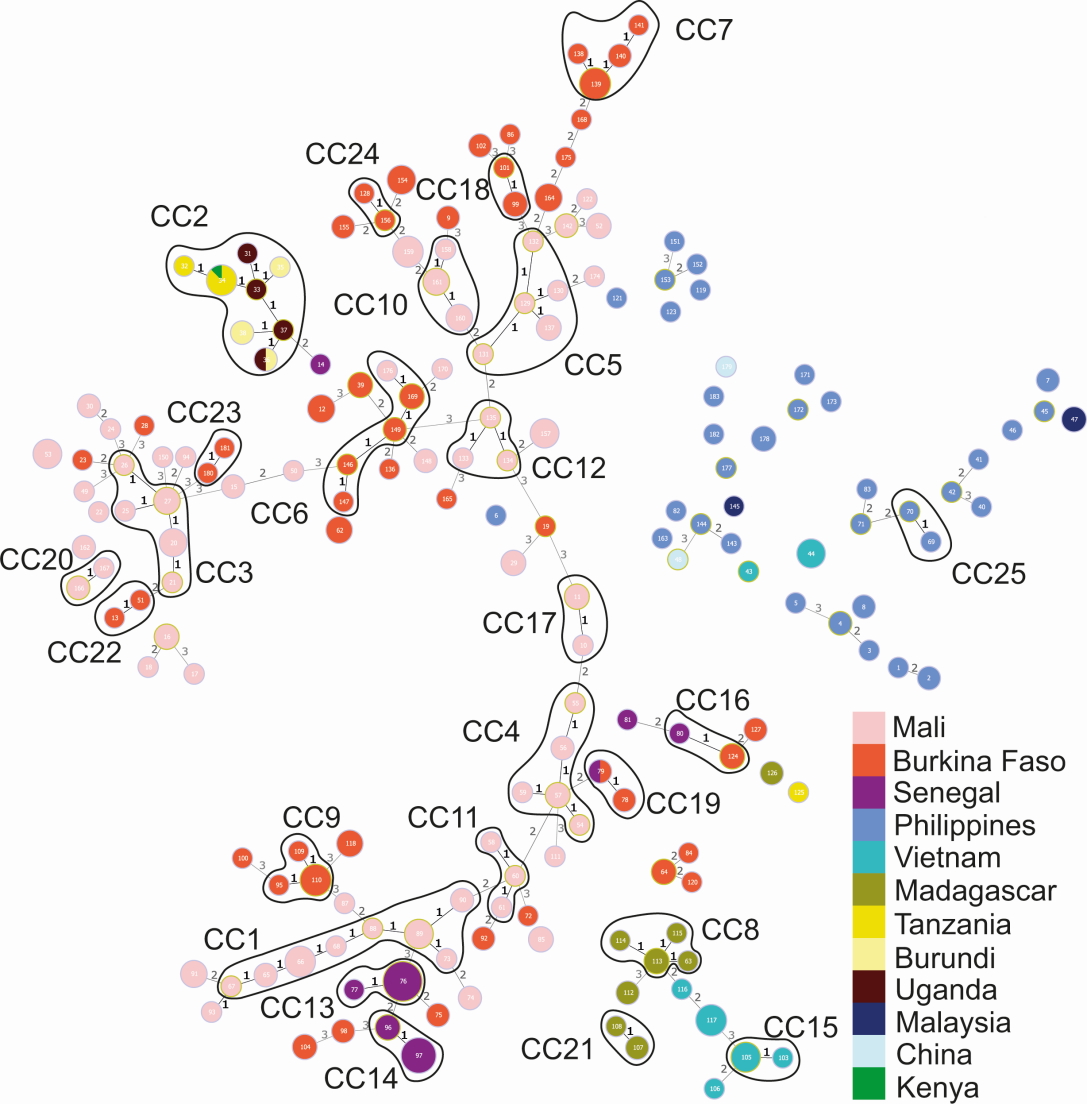
Minimum spanning tree of the *Xoc* worldwide collection. Each color corresponds to a given country. Each circle corresponds to a haplotype, and its size is log-correlated to the number of strains it contains. The haplotype number is indicated inside the circle. The numbers between haplotypes indicate the numbers of single locus differences. CCs grouping together haplotypes differing by a single locus are circled in black. Haplotypes differing by more than three loci are not linked together.

Using the MLVA-12 scheme, we identified 183 haplotypes among the 399 strains assayed. The epidemiological relationships among these haplotypes were visualized using a minimum spanning tree (Fig. 2). Eighty-three haplotypes clustered within 25 CCs, which are groups of haplotypes differing at only a SLV. These CCs may represent epidemiologically meaningful groups, corresponding to patterns of evolutionary descent. The number of haplotypes within each CC ranged from 0 to 2, and 21 of the 25 CCs consisted only of haplotypes isolated in the same country (Fig. 2). Four CCs included haplotypes of strains isolated in different countries. CCs 6, 16, and 19 each contained strains from two of the three neighboring WA countries, Mali, Burkina Faso, and Senegal, supporting the hypothesis that *Xoc* may spread across borders within this region. Several double-locus variants, that is, strains differing at two of the 12 loci, also occurred among strains from these WA countries, reinforcing the idea that strains from this region are epidemiologically linked. Strains from CC2 were distributed across four neighboring continental East African countries, including Tanzania, Burundi, Kenya, and Uganda, which supports epidemiological links among these countries. Asian strains, originating mainly from the Philippines, were either not represented in a CC or were in a CC with only strains from the same country, probably due to the low number of strains genotyped in this study from these highly genetically diverse areas. Finally, when considering the 41 strains analyzed by both sequencing and MLVA, it is evident that strains in different subclades as defined in the sequence-based phylogenetic analysis are never in the same CC or group of double locus variants as defined by the MLVA (Fig. 1 vs Fig. 2). The same clade or subclade may, however, include individuals with up to three locus differences.

Next, to determine whether populations in different countries were genetically differentiated, we computed pairwise *F*_ST_ and *R*_ST_ values between populations. *R*_ST_ is a *F*_ST_ analog that assumes a stepwise mutation model believed to reflect more accurately the mutation pattern of microsatellites (33, 34). All pairwise *F*_ST_ (not shown) and *R*_ST_ (Table 1) showed large population differentiations among countries in Africa (*R*_ST_ ranging from 0.261 to 0.797, *P* < 0.01) except between Burkina Faso and Mali, for which small population differentiation was observed (*R*_ST_ = 0.088, *P* < 0.01). Population differentiation was insignificant between Vietnam and the Philippines (*R*_ST_ = 0.066, *P* = 0.036) or Vietnam and Madagascar (*R*_ST_ = 0.051, *P* = 0.135) when computing *R*_ST_ (Table 1). Extending our analyses to populations from sub-regions across Mali and Burkina Faso, we found that most of them were highly significantly differentiated from one another (*P* < 0.01). Of the 21 pairwise comparisons, five showed moderate differentiation, three of which were between regions from the two different countries (Table 2). Only the populations from Sikasso and Segou, two neighboring sub-regions in Mali, were not differentiated (*P* = 0.106).

**TABLE 1:** Genetic population differentiation among countries^*a*^

	Burkina Faso	East Africa	Madagascar	Mali	Philippines	Senegal	Vietnam
Burkina Faso		<0.01	<0.01	<0.01	<0.01	<0.01	<0.01
East Africa	0.283		<0.01	<0.01	<0.01	<0.01	<0.01
Madagascar	0.654	0.639		<0.01	<0.01	<0.01	0.135
Mali	0.088	0.261	0.515		<0.01	<0.01	<0.01
Philippines	0.609	0.438	0.152	0.545		<0.01	0.036
Senegal	0.437	0.797	0.792	0.345	0.588		<0.01
Vietnam	0.613	0.473	0.051	0.507	0.066	0.704	

^*a*^ *R*_ST_ values are reported on and below the diagonal, while the corresponding *P*-values are indicated in grey above the diagonal. East Africa refers here to mainland East Africa (excluding Madagascar).

**TABLE 2:** Genetic population differentiation among regions of Burkina Faso and Mali^*a*^

		Burkina Faso				Mali		
		Boucle du Mouhoun	Cascades	Center Est	Hauts-Bassins	Koulikoro	Segou	Sikasso
Burkina Faso	Boucle du Mouhoun		<0.01	0.014	0.022	<0.01	<0.01	0.019
	Cascades	0.099		<0.01	<0.01	<0.01	<0.01	<0.01
	Center Est	0.132	0.253		<0.01	0.019	<0.01	0.026
	Hauts-Bassins	0.068	0.157	0.312		<0.01	<0.01	<0.01
Mali	Koulikoro	0.162	0.267	0.116	0.251		<0.01	<0.01
	Segou	0.112	0.131	0.176	0.157	0.10247		0.106
	Sikasso	0.078	0.122	0.103	0.144	0.128	0.024	

^*a*^ *R*_ST_ values are reported on and below the diagonal, while the corresponding *P*-values are indicated in grey above the diagonal.

### *Xoc* genetic diversity

As previously reported by Poulin et al. (17), two of the 12 VNTR loci were monomorphic across all *Xoc* strains, while the other 10 loci were polymorphic across strains from different countries and, most of the times, strains within the same country. Four of these polymorphic loci displayed three to five alleles, while the six others displayed 11 to 28 alleles. The mean Nei’s gene diversity index (*H*_*E*_) across the 10 polymorphic loci was 0.38.

Consistent with the fact that most strains from the Philippines were not closely linked together (Fig. 2), suggesting a very diverse population, the strains from the Philippines displayed both the highest genotypic richness (eMLG) using a rarefaction method to correct for the uneven sample size between countries as well as one of the highest genotypic and gene diversities (Table 3). Interestingly, high genotypic richness and diversity were also evident in strains from both Burkina Faso and Mali. Similarly, the highest allelic richness, that is, the mean number of alleles calculated using a rarefaction method, was observed in strains from the Philippines followed by Burkina Faso and Mali (Table 4). Strains from the Philippines also displayed the highest private allelic richness, that is, alleles present in only one subpopulation, for most of the loci (Table 4). Genotypic evenness ranged from 0.62 (East Africa) to 0.92 (Philippines), indicating that in none of the analyzed countries is a single bacterial predominant genotype (Table 3). In Madagascar and in the Philippines, the different genotypes observed were in close to equal abundance, while in East Africa and Senegal, abundance of genotypes was more unequal. The latter is probably due to sampling bias, since the 57 Senegalese isolates were collected from only 13 plants.

**TABLE 3:** Genotypic richness, evenness, and diversity of *Xoc* by country^*a*^

Population	Number of individuals	Genotypic richness			Genotypic diversity			Genotypic evenness	Gene diversity
		MLG	eMLG	SE	Shannon-Wiener index	Stoddart and Taylor’s index	Simpson’s index	E.5	Nei’s unbiased gene diversity
Burkina Faso	106	49	11.23	1.14	3.62	28.09	0.96	0.75	0.46
East Africa	18	9	7.20	0.91	1.81	4.15	0.76	0.62	0.18
Madagascar	13	8	8.00	0.00	1.99	6.76	0.85	0.91	0.37
Mali	134	65	11.69	1.02	3.91	38.53	0.97	0.77	0.47
Philippines	40	34	12.33	0.74	3.47	29.63	0.97	0.92	0.59
Senegal	57	8	3.68	1.00	1.20	2.50	0.60	0.64	0.13
Vietnam	26	7	4.99	0.94	1.56	3.98	0.75	0.79	0.43
Total	394	179	12.09	0.93	4.74	62.24	0.98	0.54	0.55

^*a*^ East Africa refers here to mainland East Africa (excluding Madagascar).

**TABLE 4:** Allelic richness (upper rows) and private allelic richness (lower rows) per locus in each sampled country using a rarefaction method^*a*^

Population	Average across loci	G06	G81	G25	G62	G55	G59	G60	G67	G09	G15	G80	G83
Allelic richness													
Burkina Faso	4.51	4.35	9.22	1.00	1.00	10.47	2.55	1.00	7.37	7.22	6.09	3.85	1.00
East Africa	2.03	1.93	1.93	1.00	1.00	2.93	1.93	1.00	2.93	4.92	1.93	1.93	1.93
Madagascar	2.42	2.00	4.00	1.00	1.00	2.00	3.00	1.00	3.00	7.00	2.00	2.00	2.00
Mali	4.36	3.81	9.25	1.00	1.00	8.32	3.17	1.00	7.46	7.47	6.68	2.46	1.65
Philippines	5.94	4.79	6.90	1.00	1.00	8.51	4.91	2.93	14.43	12.99	6.48	6.78	1.55
Senegal	1.73	1.41	2.00	1.00	1.00	2.46	1.81	1.41	1.65	3.94	2.71	1.41	1.00
Vietnam	2.44	2.00	3.00	1.00	1.00	4.51	2.00	2.00	3.75	4.26	2.00	2.75	2.00
Private allelic richness													
Burkina Faso	0.4	0.08	1.02	0.00	0.00	1.18	0.00	0.00	0.32	0.42	0.48	1.24	0.00
East Africa	0.12	0.00	0.00	0.00	0.00	0.35	0.00	0.00	0.95	0.13	0.00	0.01	0.00
Madagascar	0.37	0.00	0.10	0.00	0.00	0.00	0.00	0.00	2.07	2.23	0.00	0.00	0.00
Mali	0.45	0.00	2.91	0.00	0.00	0.39	0.01	0.00	0.17	0.82	0.54	0.35	0.18
Philippines	2.28	0.48	3.89	0.00	0.00	3.76	0.64	0.96	10.53	4.26	0.00	2.89	0.00
Senegal	0.05	0.00	0.00	0.00	0.00	0.02	0.00	0.00	0.00	0.17	0.35	0.11	0.00
Vietnam	0.17	0.00	0.00	0.00	0.00	0.47	0.00	0.00	0.96	0.59	0.01	0.01	0.00

^*a*^ East Africa refers here to mainland East Africa (excluding Madagascar).

### Persistence and spread of the pathogen in West Africa

**Fig 3:**
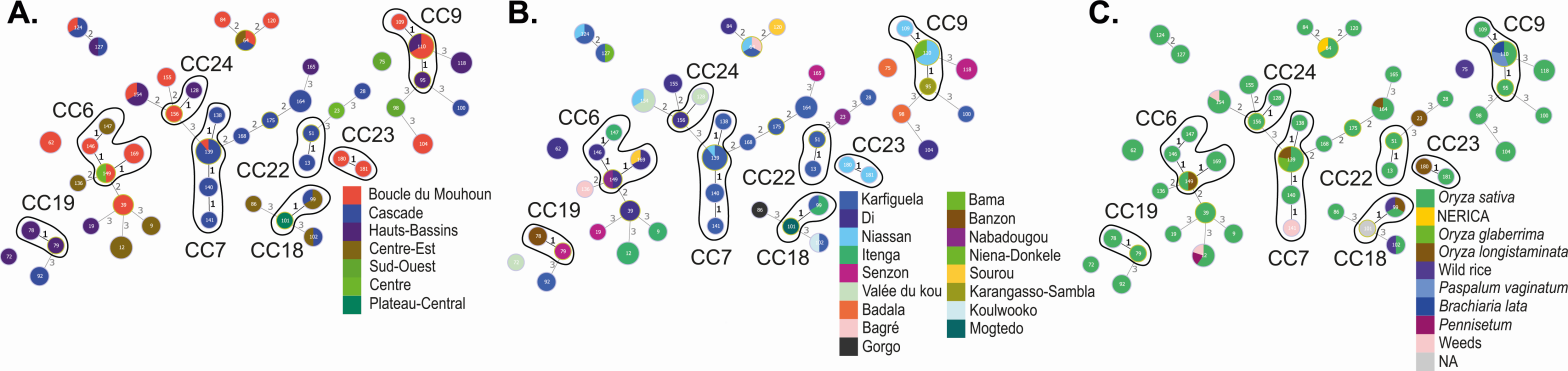
Persistence and propagation of *Xoc* inoculum in West Africa: the example of Burkina Faso. Haplotypes are colored based on their region of origin (**A**), their town or village of origin (**B**), or the host plant species from which they were isolated (**C**). CCs are circled in black. Haplotypes differing by more than three loci are not linked together.

The high genotypic diversity found in both Mali and Burkina Faso was evident even at smaller scales (Table 3). In Burkina Faso, this high diversity was found even at the scale of a region (Fig. 3A) or a village (Fig. 3B) where multiple haplotypes at the same site were not always closely related or recently diverged. At the same time, in all three WA countries, we found both single haplotypes and CCs present across at least two different sampling sites belonging to different villages/cities or even different regions, as exemplified in Burkina Faso (Fig. 3A and B). In some cases, these sites were geographically close but not always (e.g., 609 km apart between Koulwoko and Karfiguéla, Burkina Faso). A similar pattern was observed in both Mali (Fig. S4) and Senegal (Fig. S5). This suggests movement of *Xoc* between different cities and regions. The multilocus LD index *r*_*d*_ calculated for eight populations of different localities from Mali and Burkina Faso (*n* ≥ 10) was significant both when estimated from uncorrected (*P* = 0.001) and whenever possible clone-corrected data sets. Two Senegalese populations sampled from two different fields also showed a significantly high LD (Table S4). The index *r*_*d*_ was also significant for the populations at the country level (*r*_*d*_ *=* 0.315, *P* = 0.001) (Table S4).

In addition to the spatial analysis presented above, we examined whether a given genotype could be maintained in the same environment across several years. We found four haplotypes in Burkina Faso (haplotypes 62 and 139) and Mali (haplotypes 78 and 116) that were present at the same sampling location 2 consecutive years. This low number of haplotypes conserved across years in the same field is biased by the fact that only five sites were sampled at least 2 consecutive years with only one to 10 plants sampled in each case. In addition, two haplotypes (27 and 29) were isolated at the same Malian sites 6 years apart (Table S1). These results suggest either persistence of the inoculum in the field area across time or its reintroduction in or on seed collected from infected plants the prior season.

In order to determine whether plant species other than the cultivated rice species might contribute as reservoirs to the persistence of *Xoc* in the field, two cultivated rice species along with wild rice species and other weeds were included in our sampling. *Xoc* was isolated from both cultivated rice species *O. sativa* (*n* = 321 isolates) and *O. glaberrima* (*n* = 4). Within *O. sativa*, 47 different cultivars were infected. *Xoc* was also isolated from leaves of wild rice species such as the seasonal *Oryza barthii* (*n* = 11) and perennial *Oryza longistaminata* (*n* = 24) as well as three other species of weeds genetically more distantly related, such as *Brachiaria lata* (*n* = 2), *Paspalum vaginatum* (*n* = 3), and *Pennisetum* (*n* = 1). Strikingly, these different plant species, cultivated and wild rice and weeds, harbored the same bacterial haplotypes (e.g., haplotypes 21, 65, 80, 88, 101, 109, 115, and 162 on Fig. 3C) or strains related by descent (Fig. 3C; Fig. S4C and S5C). A number of these haplotypes shared among plant species were found at the same sampling site and the same year of collection in the three countries.

### Plant coinfection by distinct strains of *Xoc* occurs in West Africa

**Fig 4:**
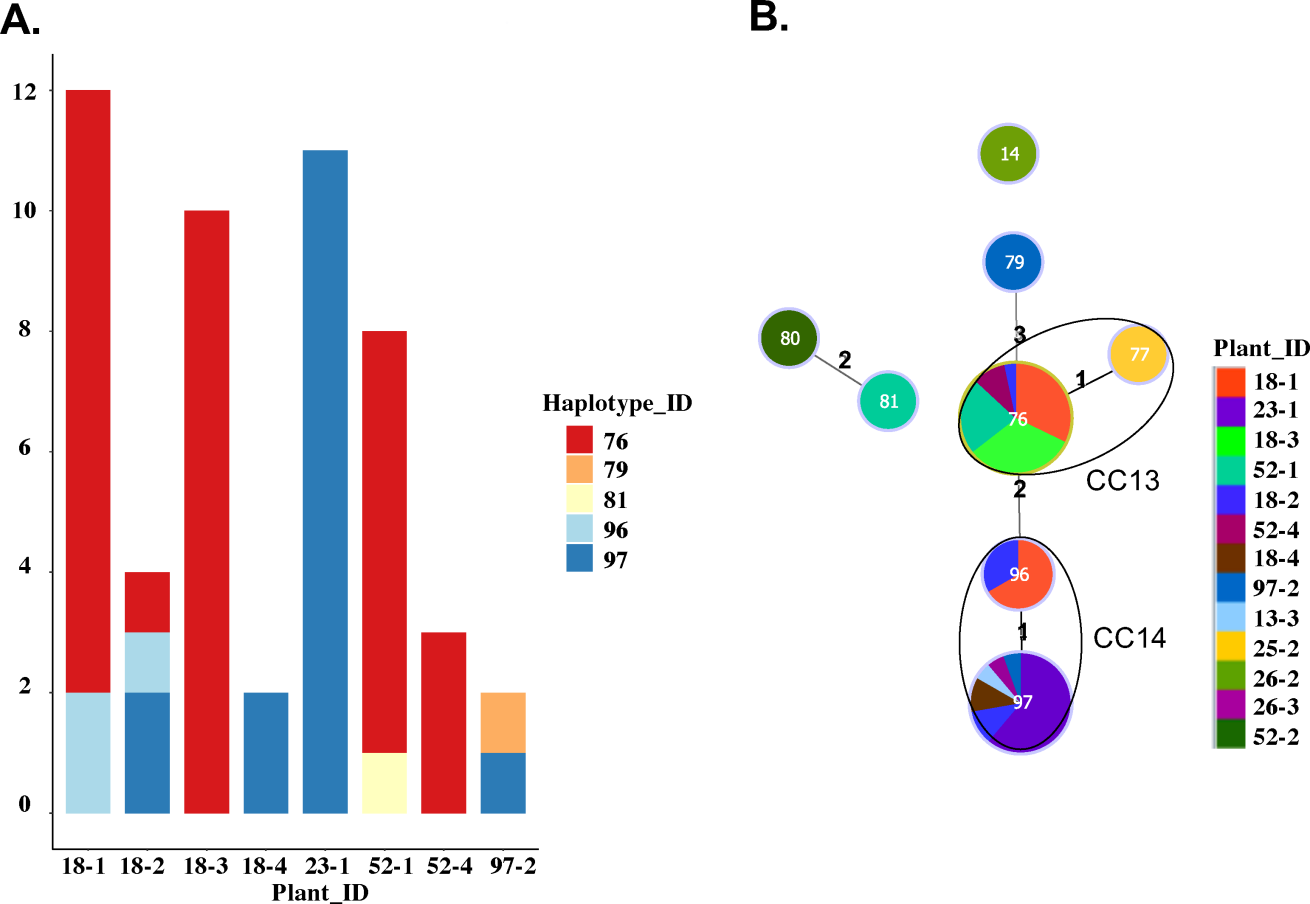
Prevalence of *Xoc* plant coinfections in Senegal. (**A**) Barplot representing the number of haplotypes found in the plants for which at least two colonies were genotyped. (**B**) Each circle represents a haplotype, with the number of the haplotype indicated inside the circle, the size of the circle reflecting the number of times it was isolated, and the color(s) indicating the individual plant(s) from which the haplotype was isolated. The key at bottom right provides for each plant the site ID (before the hyphen) and the plant ID (after the hyphen). The numbers between haplotypes indicate the numbers of single locus differences. Haplotypes differing by more than three loci are not linked. Only a single *Xoc* colony each from plants 13-3, 25-2, 26-2, 26-3, and 52-2 was genotyped, whereas between two and 12 colonies from the eight other plants were genotyped.

The large number of haplotypes found at a single sampling site, for example, in Karfiguela in Burkina Faso (Fig. 3B), prompted us to examine whether plants could be coinfected by distinct strains of *Xoc*. The possibility of coinfection is of interest as close association of distinct strains could contribute to the emergence of new genotypes by horizontal gene transfer and recombination (35, 36). Of the 399 strains analyzed by MLVA in our collection, two colonies per plant were genotyped from samples collected in different regions of Burkina Faso between 2017 and 2018. This allowed us to investigate coinfections of one plant by multiple haplotypes. While six of these samples gave colonies with the same haplotype, one plant was found to be coinfected by two haplotypes differing by five loci (Table S3). A similar situation was observed in samples from Mali, where the haplotypes of two to six colonies were obtained from each of six plants in two different regions. Five of them were found to be coinfected, four by two haplotypes that differed by at least four loci, and one by two haplotypes differing by a single locus (Table S3). Finally, in Senegal, where 13 plants were sampled, and two to 12 colonies isolated and genotyped for eight of them (Fig. 4; Table S3), four of the eight plants were found to be coinfected by two to three haplotypes each (Fig. 4A). Interestingly, of the eight total haplotypes identified in Senegal, two were found more frequently than any others, suggesting an overall high prevalence: number 76 was found in five of the 13 total plants sampled in two localities, three of these being co-infected plants, and number 97 was found in six plants isolated in four localities, two of them being coinfected (Fig. 4A; Table S3).

## DISCUSSION

Studying the genetic diversity and population structure of an organism can help reconstruct its evolutionary and demographic history. When applied to pathogenic microorganisms, it may also be an important source of information for epidemiological surveillance and can contribute to the adoption of appropriate control methods to prevent pathogen spread. Population studies usually rely on molecular markers that have different mutation rates, the choice of the molecular marker being used depending on the spatial and temporal scale under study (35). For instance, slow-evolving markers such as single-nucleotide polymorphisms (SNPs) are more appropriate for studying populations over greater distances and time (37), while fast-evolving markers such as VNTRs usually have higher discriminatory power and are more suitable for local epidemiology. We used both SNPs (core-genome alignment) and VNTRs (MLVA) to shed light on the global and local diversity and population structure of *Xoc* with a focus on West Africa. The deep phylogeny based on the SNP markers, which are evolutionary stable, allowed us to identify three major phylogenetic divisions or clades. We confirmed the existence of an Asian clade and a WA clade, as previously reported using markers covering very small portions of the genome (13, 38, 39). By substantially increasing the number of sequenced East African strains, we also confirmed the existence of a third branch, the EA-A clade, which includes strains from East Africa, Madagascar, and Asia (15). SNPs further separated well-supported subclades within each clade. The phylogenies resulting from the use of both types of markers on a representative subset of our worldwide collection were highly congruent. Only a couple of poorly supported differences in the relationships between major clades were observed, alongside minor differences within the major clades. Broadening of the MLVA to our entire collection of isolates revealed evolutionary relationships among haplotypes found mostly within the same country or in neighboring countries, as expected for these fast-evolving markers. While the SNP-based phylogeny was more robust and showed a better resolution than the MLVA-based one, in line with other comparative studies carried out on other bacterial species on a global scale (40–43), an SNP typing approach has not yet been implemented for this monomorphic pathovar. The newly sequenced genomes in this study are a step toward the development of such an approach.

By combining MLVA genotyping of a large collection and whole-genome sequencing of a subset of that collection, we were able to shed light on the history, spread, and persistence of *Xoc*. First, the high genetic and haplotypic diversities observed in the worldwide minimum spanning tree point toward *Xoc* being endemic in the Philippines. This was previously suggested by a study examining the genetic diversity among 123 Philippines strains (18, 44). This is also supported by the high private allelic richness observed in the Philippines. However, we note that strains from the Philippines were isolated between 1981 and 2009 and originated from numerous localities or regions. Second, the position of the two sequenced Philippines strains in the phylogeny is in line with an introduction of *Xoc* from this country into the other Asian countries represented. A larger number of sequenced strains from tropical and subtropical Asia would nonetheless be needed to confirm this hypothesis. Third, the subdivision of the EA-A clade into three well-supported sub-clades, two of which contain Asian strains, suggests that the bacterium was introduced from Asia into East Africa on several occasions. This could be explained through the importation of infected seeds of different *O. sativa* cultivars, which may have started around the 8th to the 10th centuries (45). Our geography-based ancestral state reconstruction analysis further supports an introduction from Asia to East Africa.

The picture for West Africa is less clear. Although 13 new strains from West Africa were sequenced for this study, the distinct grouping of all WA strains apart from strains from other areas makes inference of the history of *Xoc* in this region challenging. However, the high genetic diversity observed in Burkina Faso and Mali, in line with a previous report (16), suggests either that *Xoc* has been introduced several times into these countries or that the outbreaks in West Africa are the result of an old introduction event followed by diversification. As shown previously, our data indicate that recent signatures of descent exist between WA and Asian strains (13, 16, 20, 38). Our data support movement between WA countries with several genetic signatures indicating exchanges, probably via infected plant material such as seed. To account for the existence of a group of *Xoc* strains specific to West Africa and this high genetic diversity, Wonni et al. (16) suggested that some populations of *Xoc* could be endemic in West Africa. The position of the WA clade in our ML phylogeny and the results from our ancestral state reconstruction analysis do not support this scenario. Rather, while our genomic analysis confirms the existence of a WA clade, a region where the disease was first reported 40 years ago (44), it also shows common ancestry with the EA and EA-A clades, and the variation observed in the MLVA is consistent with clonal evolution with frequent bottleneck and genetic drift having created considerable diversity following an initial introduction from Asia. The diversity may have been increased further by more recent re-introduction and/or by exchanges and recombination within the region.

The intensification of rice cultivation in West Africa over the last decades along with the continued importation of rice seeds from Asia and the absence of rice cultivars resistant to BLS may have led to unimpeded spread and evolution of the pathogen population in the region, explaining the high genetic diversity observed. The 2008 food price crisis, especially, has driven increases in rice production and contributed to the growing importance of rice as a staple food in WA countries. Increased production has relied mostly on expanding rice cultivated areas (1). WA governments have boosted rice production by deploying improved rice varieties and distributing fertilizers (1). The trade, either formal or informal, of rice seeds for which phytosanitary regulations are still ineffective across the Economic Community of West African States (ECOWAS) (1) may have favored the dissemination of the pathogen within and between these countries. This is supported by the presence of the same bacterial genotypes in several geographically distant fields, villages, or even regions. Seed trade between neighboring countries such as Mali and Burkina Faso may explain the weak genetic differentiation observed between strains from these two countries. Interestingly, while Senegal is also part of the ECOWAS, our results highlight great *Xoc* population differentiation between this country and Mali and Burkina Faso. Further sampling might explain this observed differentiation and the origins of the different populations. Sampling in other WA countries will also be important, including in Ghana and Côte d’Ivoire to which the Burkinabe seed company NAFASO exports rice seeds (1). Sampling efforts in those countries will determine whether *Xoc* is disseminated via infected seeds.

A different epidemiological situation was observed in East Africa, where one CC grouped most of the isolates from the region, with the exception of the Malagasy ones. A more recent and less extensive sampling effort (18 isolates collected in 2013 and 2016 in “mainland” East Africa, as opposed to the 297 isolates spanning 17 years in West Africa) may explain the lower diversity in East Africa. It will be important to analyze a greater number of strains from East Africa. Nonetheless, our results suggest (i) a common origin or repeated exchange of inoculum between countries of continental East Africa and (ii) a highly probable Asian origin for strains from East Africa and Madagascar.

While seed trade likely plays a major role in *Xoc* long-distance propagation, our results also support the role of alternative hosts in its persistence and as a source for short-distance spread. Although wild rice species and weeds have been previously reported as hosts of *Xoc* in West Africa (16), our results revealed infections of these hosts by the same bacterial genotypes as the cultivated rice species, supporting their roles as inoculum reservoirs. For instance, the irrigation channels constantly invaded by wild rice in Senegal may contribute to the persistence and short-distance dissemination of the pathogen. We sampled a limited number of such species. A systematic sampling of all weeds found within or close to *Xoc*-infected rice fields may reveal additional potential alternative hosts contributing to *Xoc* epidemics. Systematic genotyping of weed isolates would further inform the population genetic structure of *Xoc* and management strategies to reduce inoculum pressure and spread of the disease. Regarding cultivated rice, we found that *Xoc* not only infects the Asian cultivated rice species *O. sativa* but also the African rice species *O. glaberrima*, which is usually more resistant to pathogens (4). The fact that several weeds, wild rice, and cultivated rice species harbor *Xoc* likely contributes to co-infection of plants by distinct *Xoc* strains, which we observed in both the cultivated rice species *O. sativa* and the wild rice species *O. longistaminata*, by supporting both *Xoc* prevalence and genetic diversity. Although we investigated co-infections on a limited number of plants, our results reveal the potential importance of this phenomenon, with 71.4% (5/7) of plants sampled in Mali and 50% (4/8) of those in Senegal yielding two or more *Xoc* haplotypes. Furthermore, analysis of the haplotypes found within these coinfected plants suggested that in most cases, they were not evolutionary related, suggesting independent infection events. We detected fewer instances of coinfection in Burkina Faso (14%), but this could be due to the lower number of colonies per plant (*n* = 2) genotyped, bacterial genotypes being not necessarily at the same relative frequency within an infected plant. This possibility is supported by some of the coinfected samples from Mali and Senegal, from which a given genotype was isolated at a higher relative frequency. Importantly, co-infection may allow for genetic exchanges between strains, potentially leading to the emergence of novel genotypes. We did not find any evidence for extensive recombination in the *Xoc* populations. The two different sets of genetic markers used in this study with different levels of discrimination have produced convergent phylogenies from a collection of Asian and African *Xoc* which were highly congruent. This clear phylogenetic signal complementary to the strong LD observed at the country, locality, and field level supports a predominant clonal evolution (46). In line with this, we observed the presence of discrete genetic subdivisions at the pathovar level and the occurrence of several CCs, including groups of single- and double-locus variants (45). However, a deeper sampling at the field or at the plant level where co-infections may occur will enable better exploration of the population structure and estimation of the importance of recombination in the evolution of *Xoc*. Recombination might be an important source of variability for some genomic regions in particular, including those associated with virulence. For instance, previous studies in *X. oryzae* pv. *oryzae* suggested that recombination events may contribute to the fast evolution of TAL effectors and T6SS-related genes (27, 47–49).

To conclude, this study brings a much-needed understanding of both the local and global factors coming into play in the persistence and propagation of *Xoc*. By detailed analysis of a large collection of strains, we were able to discern how this pathogen spreads across different regions. Two likely factors have been highlighted: the role of contaminated seeds in short- and long-distance dissemination as a result of uncontrolled seed trading and the role of plant reservoirs in the persistence and spread of the disease. The strong evidence for these factors suggests key measures that could be taken to help limit the spread and impact of the disease, particularly in Africa, namely, seed certification and weed control. Specifically, while the international trade of rice seeds between countries, notably between Asia and Africa, may be at the origin of the *Xoc* outbreaks in the latter and the enforcement of phytosanitary surveillance for import from Asia could help stem re-introductions, enforcement of such legislation within WA states might contribute to a decrease in the incidence and spread of *Xoc* and other seed-transmitted pathogens infecting rice and thus sustain the much-needed rice production there. Programs to encourage a targeted control of *Xoc* alternative hosts surrounding rice fields may further decrease the inoculum pressure.

## SUPPLEMENTAL MATERIAL

Fig. S1 aem.01121-25-s0001.tif Geographic ancestral reconstruction of *Xoc*.

Fig. S2 aem.01121-25-s0002.tif Tanglegram of the MLVA-based and core genome-based trees.

Fig. S3 aem.01121-25-s0003.tif Rarefaction curve of total genotypes according to the number of loci sampled.

Fig. S4 aem.01121-25-s0004.tif Persistence and propagation of *X. oryzae* pv. *oryzicola* inoculum in Mali.

Fig. S5 aem.01121-25-s0005.tif Persistence and propagation of *X. oryzae* pv. *oryzicola* inoculum in Senegal.

Supplemental tables aem.01121-25-s0006.xlsx Tables S1 to S4.

ASM does not own the copyrights to Supplemental Material that may be linked to, or accessed through, an article. The authors have granted ASM a non-exclusive, world-wide license to publish the Supplemental Material files. Please contact the corresponding author directly for reuse.
