# Supplementary figures and images for: Large- and small-scale population structure of Xanthomonas oryzae pv. oryzicola, a bacterial pathogen of rice
Source: Appl Environ Microbiol. 2025 Sep 8;91(10):e01121-25. doi: 10.1128/aem.01121-25 (PMC12542787; doi:10.1128/aem.01121-25)

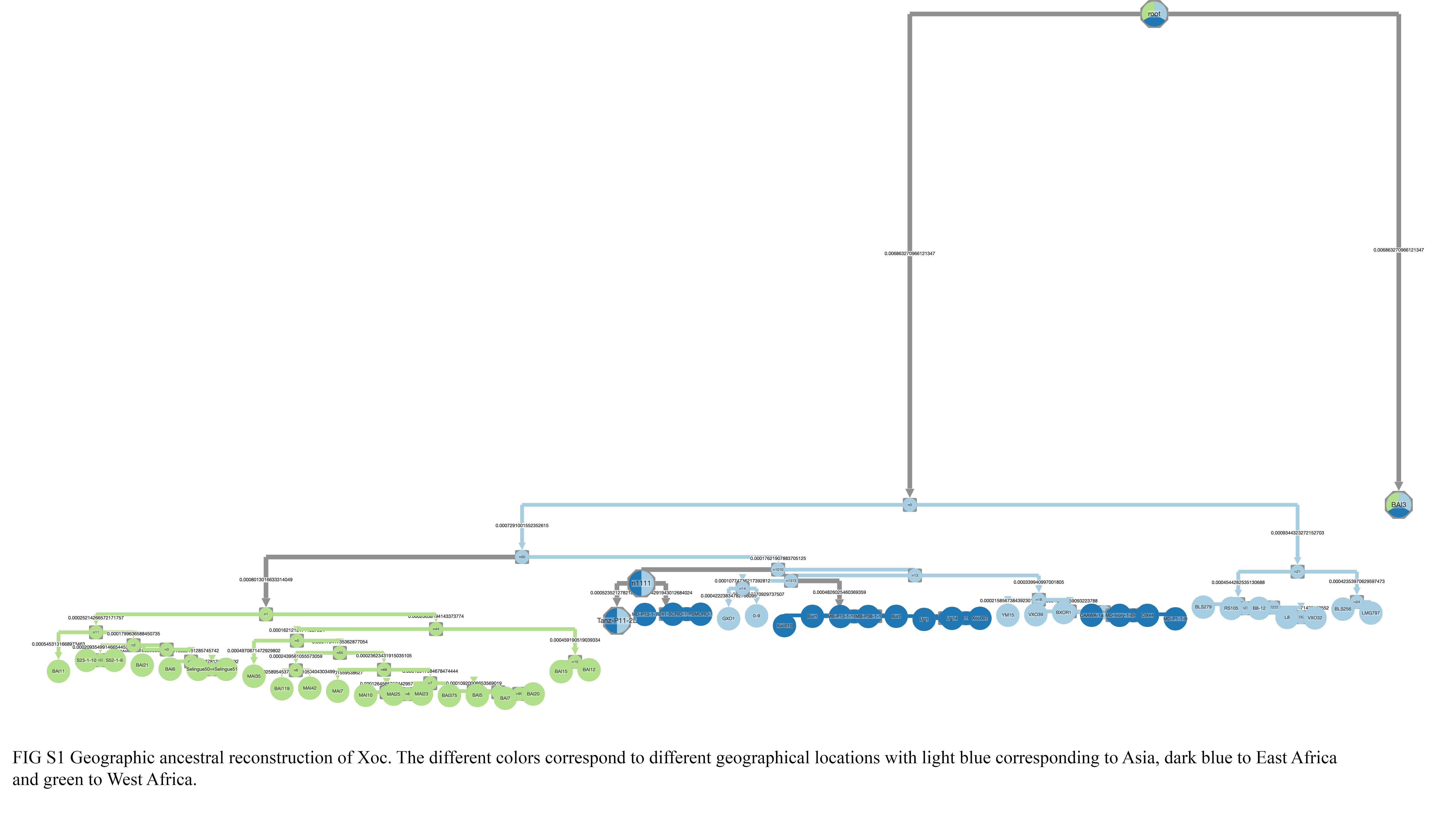

Supplement: Fig. S1 — Geographic ancestral reconstruction of Xoc. [file aem.01121-25-s0001.tif]

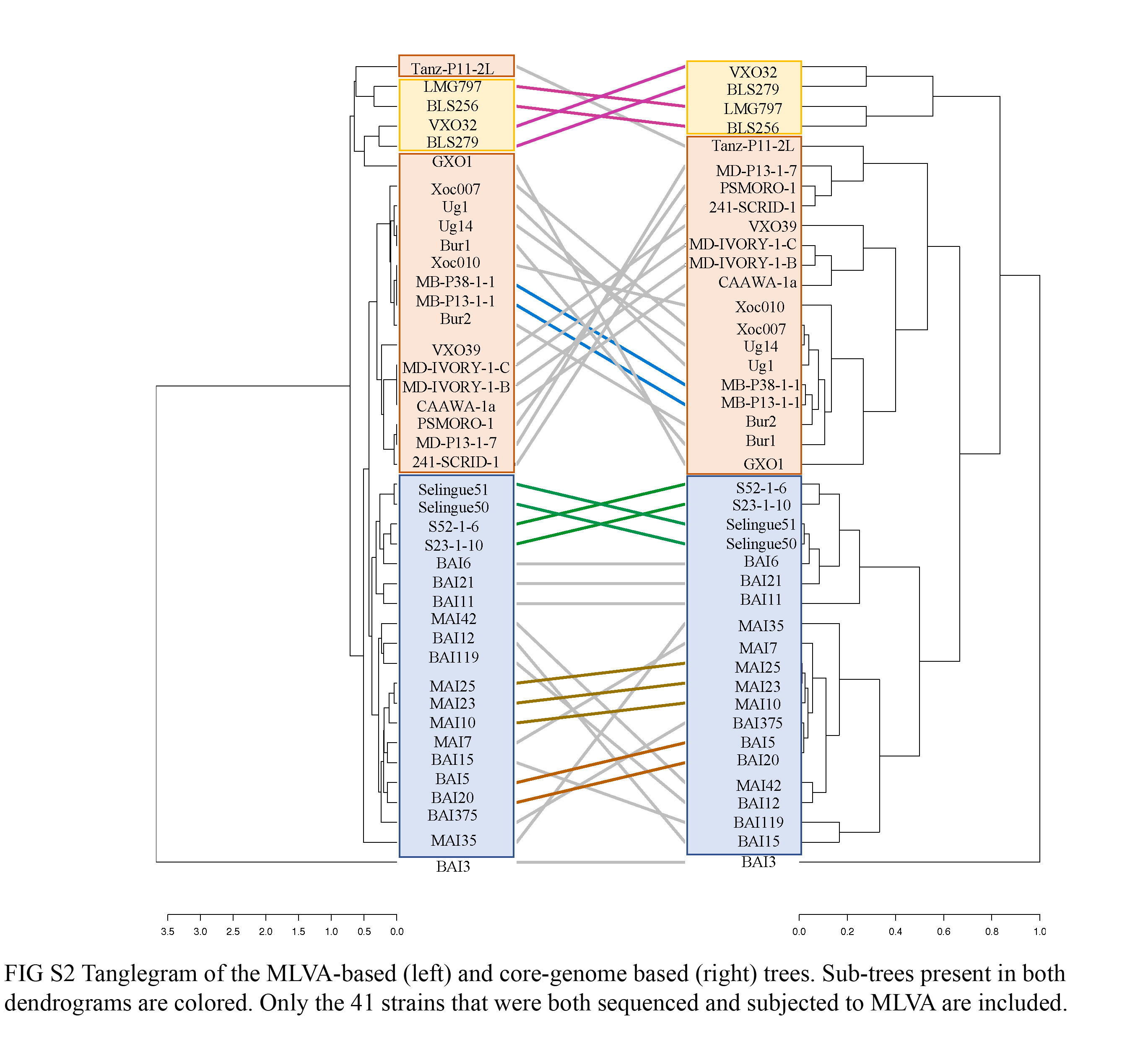

Supplement: Fig. S2 — Tanglegram of the MLVA-based and core genome-based trees. [file aem.01121-25-s0002.tif]

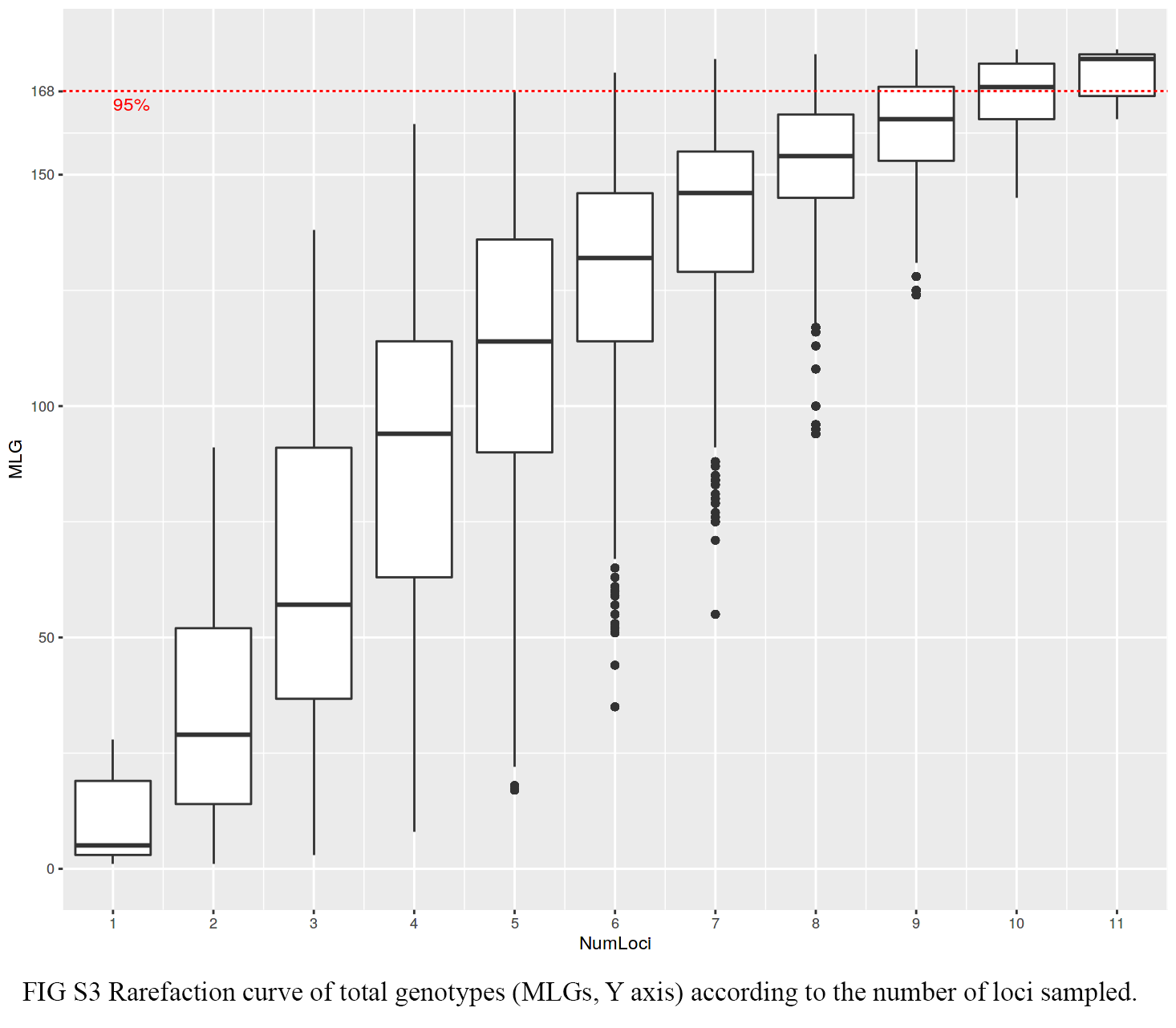

Supplement: Fig. S3 — Rarefaction curve of total genotypes according to the number of loci sampled. [file aem.01121-25-s0003.tif]

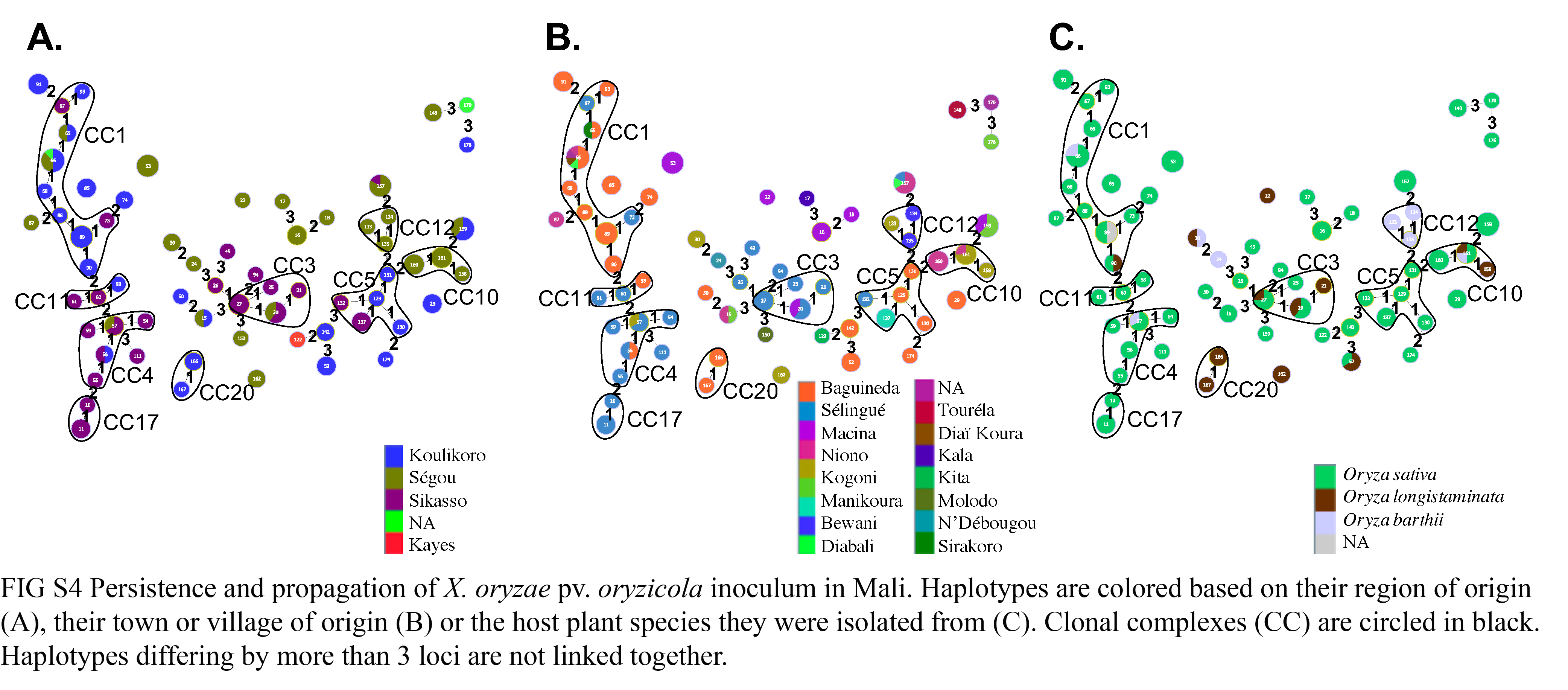

Supplement: Fig. S4 — Persistence and propagation of X. oryzae pv. oryzicola inoculum in Mali. [file aem.01121-25-s0004.tif]

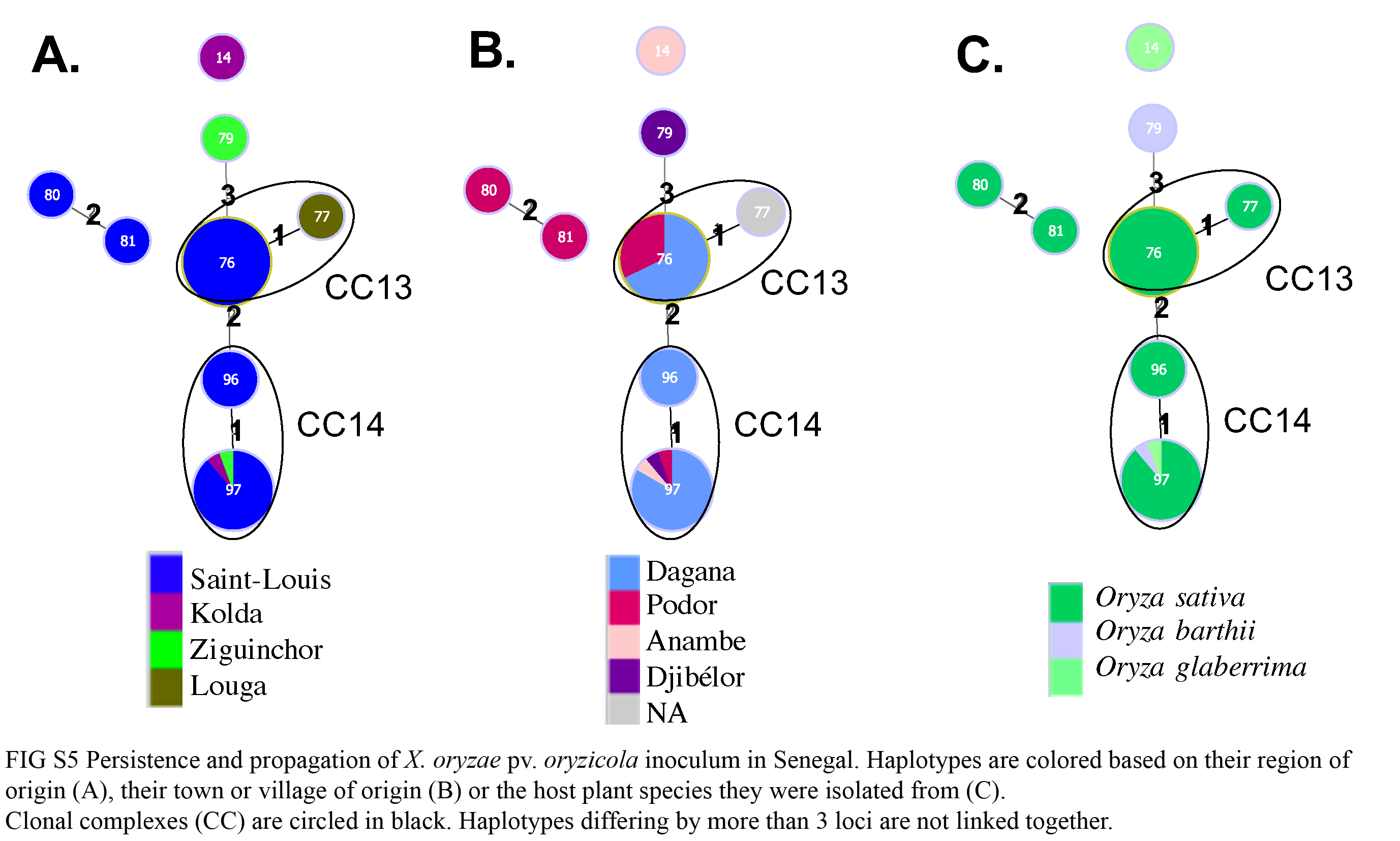

Supplement: Fig. S5 — Persistence and propagation of X. oryzae pv. oryzicola inoculum in Senegal. [file aem.01121-25-s0005.tif]
